# Clinical Effectiveness of Protein and Amino Acid Supplementation on Building Muscle Mass in Elderly People: A Meta-Analysis

**DOI:** 10.1371/journal.pone.0109141

**Published:** 2014-09-30

**Authors:** Zhe-rong Xu, Zhong-ju Tan, Qin Zhang, Qi-feng Gui, Yun-mei Yang

**Affiliations:** 1 Department of Geriatrics, First Affiliated Hospital, School of Medicine, Zhejiang University, Hangzhou, China; 2 State Key Laboratory for Diagnosis and Treatment of Infectious Diseases, Department of Geriatrics, First Affiliated Hospital, School of Medicine, Zhejiang University, Hangzhou, China; Texas A&M University, United States of America

## Abstract

**Objective:**

A major reason for the loss of mobility in elderly people is the gradual loss of lean body mass known as sarcopenia. Sarcopenia is associated with a lower quality of life and higher healthcare costs. The benefit of strategies that include nutritional intervention, timing of intervention, and physical exercise to improve muscle loss unclear as finding from studies investigating this issue have been inconsistent. We have performed a systematic review and meta-analysis to assess the ability of protein or amino acid supplementation to augment lean body mass or strength of leg muscles in elderly patients.

**Methods:**

Nine studies met the inclusion criteria of being a prospective comparative study or randomized controlled trial (RCT) that compared the efficacy of an amino acid or protein supplement intervention with that of a placebo in elderly people (≥65 years) for the improvement of lean body mass (LBM), leg muscle strength or reduction associated with sarcopenia.

**Results:**

The overall difference in mean change from baseline to the end of study in LBM between the treatment and placebo groups was 0.34 kg which was not significant (P = 0.386). The overall differences in mean change from baseline in double leg press and leg extension were 2.14 kg (P = 0.748) and 2.28 kg (P = 0.265), respectively, between the treatment group and the placebo group.

**Conclusions:**

These results indicate that amino acid/protein supplements did not increase lean body mass gain and muscle strength significantly more than placebo in a diverse elderly population.

## Introduction

Sarcopenia is an age related loss of muscle mass and strength, and is associated with a lower quality of life resulting from a reduced ability to perform daily living tasks [1]. Sarcopenia results in increased healthcare costs of approximately $900 per elderly adult which in the USA is approximately $18.5 billion per year [2]. Prevalence of sarcopenia differs by gender, living circumstances, and continent: 13.2% of Chinese men and 4.8% of Chinese women who are ≥70 years of age have sarcopenia, while 45–70% and 7–17.5% of American men and 2%–59% and 4–10% of American women have sarcopenia, respectively [3]. Age-related muscle loss is highly prevalent in nursing homes, with rates being as high as 68% in elderly men and 21% in elderly females [4], whereas community dwelling elderly have lower prevalence rates in males (10%) but higher rates in women (33%) [5].

Inadequate nutrition, oxidative stress, low physical activity levels, inflammation, and reduced hormone concentrations contribute to age related muscle loss [6]. Possible strategies that reliably increase muscle mass and strength in the elderly have been actively investigated, but conclusions on the benefits of different nutritional interventions, timing of administration, and physical exercise from studies have been conflicting [7]–[20].

Several nutritional interventions such as creatine monohydrate, whey protein, caseinate, and essential amino acids appear to augment protein synthesis in muscles [1], [21], [22]. Numerous studies have found that these nutritional supplements enhance the magnitude of gain in lean body mass and muscle strength in older adults undergoing exercise training [1], [6], [15]. Essential amino acid and leucine supplementation have increased protein synthesis in muscles and are thought to be better strategies for offsetting muscle loss than intact protein [7], [16], [22]–[24], due in part to their higher absorption [22]. However, several studies that compared the effect of whey protein or amino acid supplementation on skeletal muscle mass, lean body mass, or strength in healthy elderly to that of placebos have not detected a significant difference between the two groups [8], [17].

Many of the studies evaluating the impact of protein or amino acid supplementation on sarcopenia have been small and evaluated different supplements. In order to maximize the biostatical power of placebo controlled clinical trials, we have performed a meta-analysis to assess the ability of protein or amino acid supplementation to augment lean body mass or strength of leg muscles in elderly patients.

## Experimental Methods

PubMed, Google Scholar, The Cochrane Library, EMBASE, and ClinicalTrials.gov were searched from inception to 13 Jun 2014 using combinations of the following terms: aging, elder, older, muscle loss or muscular atrophy, protein, amino acid. Inclusion criteria for the meta-analysis required that an article be published in a peer-reviewed reviewed journal that described a prospective study or randomized controlled trial (RCT) which compared the efficacy of an amino acid or protein supplement with placebo in improving lean body mass, leg muscle strength in elderly people (≥65 years of age). Single group uncontrolled studies, cross sectional studies, or retrospective studies were excluded. Studies published as letters, comments, editorials, or case reports were also excluded, as well as studies that included people <65 years of age. We utilized the Delphi list to assess the quality of the included studies [25].

### Data extraction

Full text articles for the relevant titles were assessed for eligibility which included studies that measured changes in lean body mass (LBM), and may have included evaluation of muscle strength of leg extension and double leg press. Two independent reviewers (coders) extracted the following information from each eligible study: cited reference, type of study, type and duration of interventions, participant number in the intervention and placebo groups, demographics of participants (age, sex, mean body mass index [BMI]), and mean values of the outcome measures (LBM, muscle strength in double leg press, muscle strength in leg extension) at baseline and post intervention. In case of a disagreement, a third reviewer resolved the issue.

To assess coder drift, agreement between coders was calculated by dividing the number of variables coded the same by the total number of variables. Mean agreement of ≥0.90 was considered to be acceptable.

### Biostatistics

Treatment effectiveness was evaluated by comparison of LBM (primary outcome) and muscle strength of double leg press and leg extension (secondary outcomes) in elderly subjects at baseline and after nutritional intervention for 6 months (24 weeks). For treatment consistency, only studies providing protein supplementation were considered for meta-analysis. The means with standard deviations (SD) for the LBM, mean muscle strength (leg press and leg extension) were calculated for each group at baseline and post study completion. The difference in mean change (from baseline to end of study) with 95% confidence interval (95% CI) was calculated as the mean change of the protein intervention (treatment group) minus mean change of the placebo or non-nutritious supplements (control group) for each outcome.

Heterogeneity was determined by calculating Cochran Q and the I^2^ statistic. The Q statistic indicated statistically significant heterogeneity at *P*<0.10. The I^2^ statistic reflected the percentage of the observed between-study variability and provided a scale of heterogeneity: 0 to 24% = no heterogeneity; 25 to 49% = moderate heterogeneity; 50 to 74% = large heterogeneity; and 75 to 100% = extreme heterogeneity. If heterogeneity existed between studies (a Q statistic with *P*<0.1 or an I^2^ statistic >50%), we performed the random-effects model (DerSimonian-Laird method). Otherwise, the fixed-effects model was recommended (Mantel-Haenszel method). Combined difference in mean change from baseline to end of study was calculated and a 2-sided *P* value <0.05 was considered to indicate statistical significance. Sensitivity analysis was performed using the leave-one-out approach. Publication bias was only assessed for lean body mass by constructing funnel plots and exacerbations rate by Egger’s test. The absence of publication bias is indicated by the data points forming a symmetric funnel-shaped distribution and one-tailed significance level P>0.05 in Egger’s test. All statistical analyses were performed using the statistical software Comprehensive Meta-Analysis, version 2.0 (Biostat, Englewood, NJ, USA).

## Results

**Figure 1:**
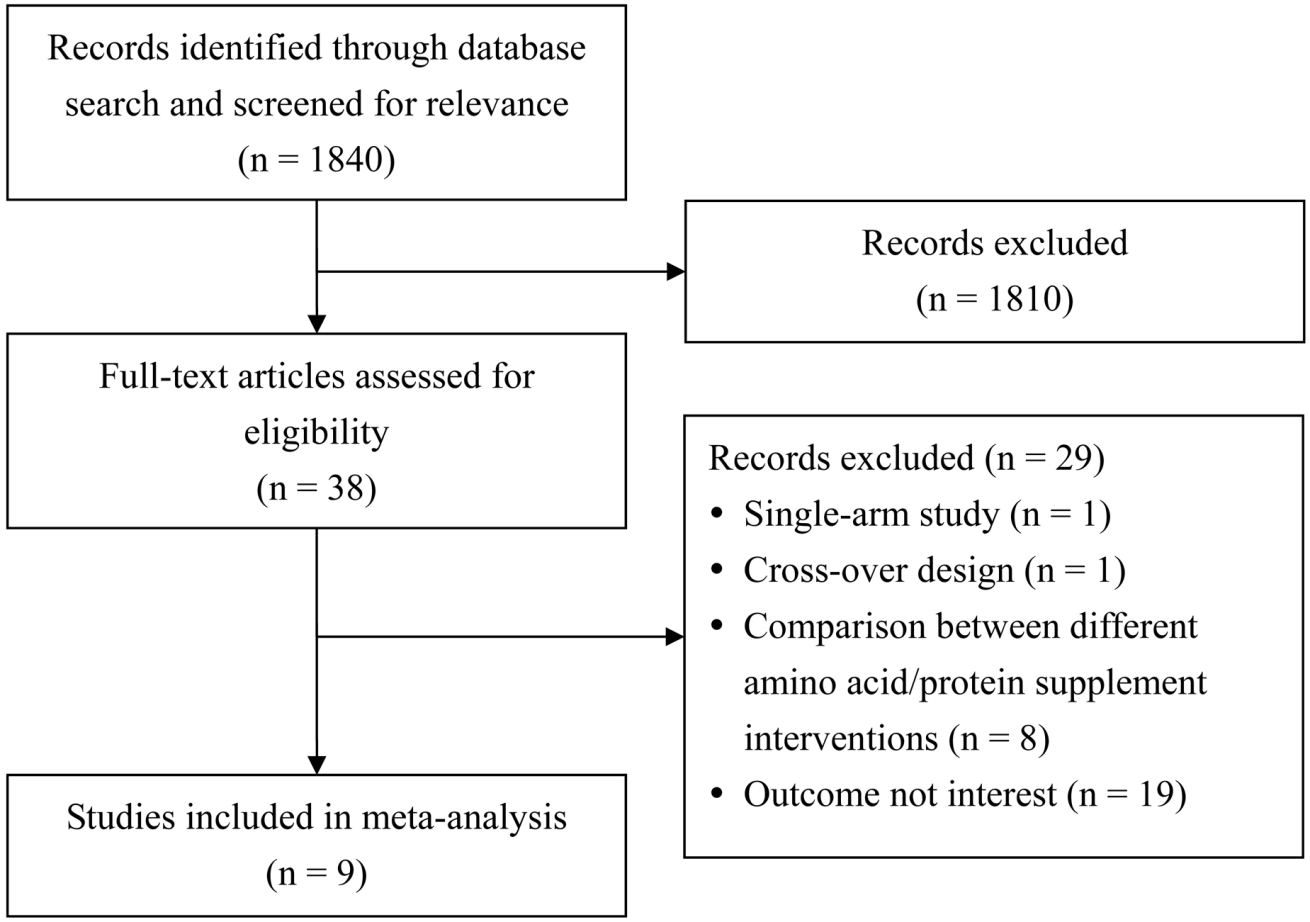
Flow diagram of study selection.

Out of 1840 studies identified by the data base searches, 38 were screened for eligibility, and 29 were excluded for one of the following reasons: no comparison group (n = 1), no placebo (n = 8), cross over design (n = 1) or no value for mean muscle mass or leg muscle strength (n = 19) (Figure 1). Nine prospective studies met the inclusion criteria (Figure 1) [8], [17]–[20], [26]–[29].

All but one of the studies [18] were at least 75% compliant with the Delphi list (Table 1). Eight of the 9 studies were randomized, placebo-controlled clinical trials [8], [17], [19], [20], [26]–[29]. Five of the trials included an intention-to-treat analysis [8], [20], [26], [27], [29]. The 75%–100% compliance levels of 8 of the 9 studies to the Delphi criteria suggest that the studies provided high quality evidence. Coder drift was calculated to be 0.93, indicating satisfactory reliability between coders.

**Table 1:** Quality assessment of the 9 studies included in the systematic review and meta-analysis as determined using the Delphi List.

Author(Year)	Was amethodofrandomization used?	Were the groups similar atbaselineregarding themost important prognosticindicators?	Were theeligibilitycriteriaspecified?	Was theoutcomeassessorblinded?	Was thecare providerblinded?	Was thepatientblinded?	Were pointestimateand measuresof variabilitypresentedfor theprimaryoutcomemeasures?	Did the analysis include an intention-to-treat analysis?
Daly et al [27]	Yes	Yes	Yes	No	No	Yes	Yes	Yes
Vermeeren et al [28]	Yes	Yes	Yes	Yes	Yes	Yes	Yes	No
Chale et al [8]	Yes	Yes	Yes	Yes	Yes	Yes	Yes	Yes
Alemán-Mateo et al[29]	Yes	Yes	Yes	Yes	No	No	Yes	Yes
Tieland et al [20]	Yes	Yes	Yes	Yes	Yes	Yes	Yes	Yes
Tieland et al [26]	Yes	Yes	Yes	Yes	Yes	Yes	Yes	Yes
Leenders et al [19]	Yes	Yes	Yes	Yes	Yes	Yes	Yes	No
Ferrando et al [18]	Yes	Gender different, otherssimilar	Yes	No	No	Yes	Yes	No
Verhoeven et al [17]	Yes	Yes	Yes	Yes	ND	Yes	Yes	No

ND, not described.

The number of total participants in all 9 studies who had taken the intervention was 267 (range, 10 to 53) and who had received placebo were 244 (range, 11 to 47). Six of the 9 studies provided a protein supplement (whey) to 203 elderly participants and placebo to 191 elderly subjects (controls) [8], [20], [26]–[29], 2 studies supplied leucine supplementation to 54 elderly participants and placebo to 42 controls [17], [19], and one provided essential amino acids (EAA) to 10 elderly participants and 11 controls [18] (Table 2). The duration of intervention ranged from 10 days to 6 months (Table 2).

**Table 2:** Characteristics of studies included in the systematic review and meta-analysis.

Author(Year)	Studytype	Comparison	Duration ofIntervention	Numberof cases	MeanAge (year)	Sex(Male %)	Mean BMI(kg/m^2^)	Lean bodymass (kg)		Muscle strength(kg), double leg press		Muscle strength (kg),leg extension	
								Before	After	Before	After	Before	After
Dalyet al [27]	RCT	Protein vsControl	4 months	53 vs 47	72 vs 74	0 v. 0	28 vs 28	0.6 (0.3, 0.8)* vs 0.1 (−0.4, 1.1)*		NA	NA	28 (18, 39)* vs 10 (−1, 21)*	
Vermeerenet al [28]	RCT	Protein vsControl	mean 9 days	23 vs 24	66 vs 65	61 vs 75	20 vs 21	Mean change from baseline: –0.5±2.6 vs –0.4±2.7		NA	NA	Mean change from baseline: 3±8 vs 2±9	
Chaleet al [8]	RCT	Protein vsControl	6 months	42 vs 38	78 vs 77.3	40 vs 42	27 vs 26.9	46.7±8.6 vs 46.4±8.4	47.3±8.6 vs 46.7±8.4	125±39 vs 128±47	151±58 vs 149±54	NA	NA
Alemán-Mateoet al [29]	RCT	Protein vsControl	3 months	20 vs 20	75 vs 77	40 vs 45	27 vs 26	37.1±6.3 vs 36.8±6.4	37.9±6.5 vs 37.6±6.4	NA	NA	NA	NA
Tielandet al [20]	RCT	Protein vsControl	24 weeks	34 vs 31	78 vs 81	41.2 vs 48.9	27 vs 26.2	45.8±9.9 vs 46.7±9.5	45.8±9.9 vs 46.6±9.5	118±47 vs 124±50	136±47 vs 139±50	57±29 vs 57±28	68±29 vs 63±28
Tielandet al [26]	RCT	Protein vsControl	24 weeks	31 vs 31	78 vs 79	35 vs 32	28.7 vs 28.2	47.2±9.6 vs 45.7±8.9	48.5±9.4 vs 45.4±8.9	124±39 vs 116±36	169±39 vs 162±41	56±17 vs 58±17	77±18 vs 79±18
Leenderset al [19]	RCT	Leucine vsControl	24 weeks	39 vs 28	71 vs 71	100 vs 100	27.4 vs 27.2	61.9±6.9 vs 62.2±6.9	62.0±6.2 vs 62.2±6.9	202±44 vs 205±37	217±50 vs 218±42	80±12 vs 88±16	84±19 vs 94±21
Ferrandoet al [18]	RCT	Amino acidvsControl	10 days	10 vs 11	71 vs 68	10 vs 50	NA	43.0±0.6 vs 46.8±1.0	42.1±0.6 vs 45.3±1.0	NA	NA	NA	NA
Verhoevenet al [17]	RCT	Leucine vsControl	12 weeks	15 vs 14	NA	100 vs 100	25.9 vs 26.3	54.6±5.8 vs 55.8±3.4	55.0±5.8 vs 56.2±4.1	170±8 vs172±6	NA	85±3 vs85±3	NA

NA, not available; RCT: randomized controlled trial.

*values are within-group mean absolutes of the change from baseline with 95% confidence intervals in parentheses.

### Lean Body Mass

**Figure 2:**
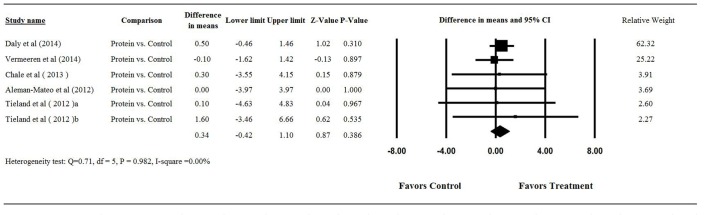
Forest plot showing results for the meta-analysis of difference in mean change from baseline in lean-body-mass after intervention: treatment vs. control. Abbreviation: CI, confidence interval.

Among the 6 studies with protein supplementation [8], [20], [26]–[29], three reported that nutritional supplementation significantly increased LBM in the elderly compared to placebo [8], [26], [27]. Two studies observed a significantly greater LBM in both the placebo and nutritional intervention groups [829]. Pooling of data from the 6 studies revealed no heterogeneity (Q = 0.71, df = 5, P = 0.982; I^2^ = 0.0%); therefore, a fixed-effects model was used to assess the difference in mean change in LBM from baseline to end of study between the placebo and protein supplementation groups. The difference in mean change of LBM from baseline to end of study between the placebo and protein supplementation groups ranged from −0.1 to 1.60 kg. The overall difference in mean change in LBM between treatment intervention and placebo was 0.34 kg which was not significant (95% CI = −0.42 to 1.10 kg, P = 0.386, Figure 2).

We compared the health status of the participants in the 9 studies to determine whether the health status of the elderly correlated with the greater gain in LBM. No significant gains in LBM compared to the controls were observed in subjects with diabetes [19], chronic obstructive pulmonary disease [29], limited mobility, who were sedentary [8], moderately active [18], or healthy and independent [17] (Table 3).

**Table 3:** Summary of 9 trials included in the systematic review and meta-analysis.

Author(Year)	Conditionof elderly	Supplementgiven	SignificantincreasedLBM tobaseline	SignificantincreasedLeg presstobaseline	SignificantincreasedLeg extensionto baseline	SignificantincreasedPhysicalperformance
Dalyet al [27]	Healthy	Max.45 gprotein/twicedaily	Significantincreased inproteingroup,differentbetweengroups	ND	Significantincreased inprotein group,differentbetweengroups	Significantincreased in bothgroup, similarbetween groups
Vermeerenet al [28]	COPD	125 ml/threetimes daily	Neithergroup	ND	Neithergroup	ND
Chaleet al [8]	Mobilitylimited	20 gprotein/daytwice daily	Both groupsimproved,and alsosignificantdifferentbetweengroups	Bothgroups tobaseline	Bothgroupsto baseline	Significant forwhey group
Alemán-Mateo et al [29]	Healthy	15 gprotein/day	Both groupsimproved,but nosignificantdifferentbetweengroups	ND	ND	ND
Tielandet al [20]	Pre-frailand frail	15 g proteintwice daily	Neithergroup	Bothgroups tobaseline	Bothgroupsto baseline	Both groups
Tielandet al [26]	Pre-frailand frail	15 gprotein twicedaily	Significantincreased inproteingroup,differentbetweengroups	Neithergroup	Trend towardsignificantimprovementin proteingroup vscontrol.	Significantimprovement inprotein group vs control.
Leenderset al [19]	Type 2diabetes	2.5 g leucinethree timesdaily	None	Increasedvs time in bothgroups,similarbetweengroups	Increased vstime in bothgroups,similarbetweengroups	ND
Ferrandoet al [18]	Moderatelyactive	15 g EAAthree timesdaily	None	ND	ND	Increased vs timein both groups,similar between groups
Verhoevenet al [17]	Healthy	2.5 g leucinethree times daily	Nonevstime orgroups	None vstime orgroups	None vstime orgroups	ND

COPD, chronic obstructive pulmonary disease; EAA, essential amino acids; LBM, lean body mass; ND, not described.

### Muscle strength: double leg press

Five of the 9 studies assessed the effect of nutritional intervention on muscle strength be double leg press [8], [17], [19], [20], [26]. Three of 5 studies reported that the strength of the leg press significantly increased in both placebo and intervention groups during the duration of the study and the mean change was similar in both groups [8]. Two studies reported no significant change in the strength of the leg press with respect to treatment time or group [17], [20].

**Figure 3:**
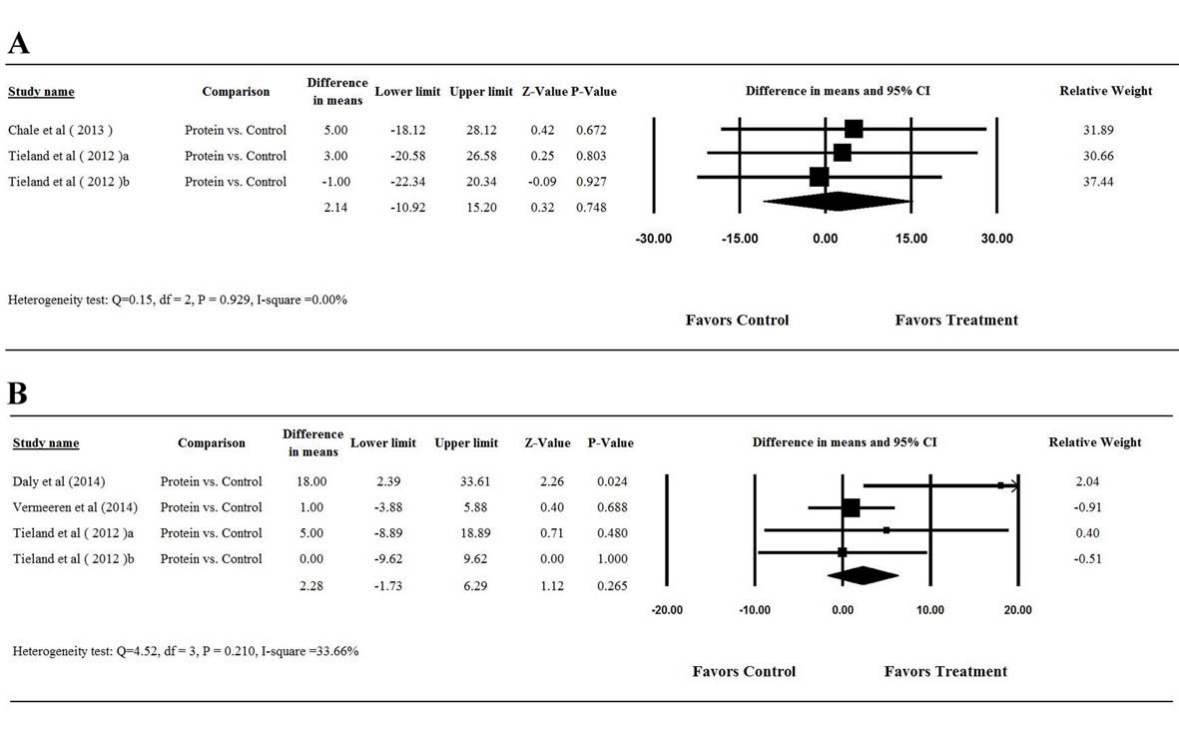
Forest plot showing results for the meta-analysis of difference in mean change from baseline in (A) muscle strength of double leg press and (B) muscle strength of leg extension after intervention: treatment vs. control. Abbreviation: CI, confidence interval.

Three studies were included in the analysis of the influence of protein supplements on leg strength [8], [20], [26]. No heterogeneity was found among 3 studies (Q = 0.147, df = 2, *P* = 0.929; I^2^ = 0.0%); and the fixed-effects model revealed no significant difference in mean change in muscle strength by double leg press between the placebo and treatment groups. The difference in mean change from baseline to end of study ranged from −1.00 to 5 kg, with the overall difference in mean change being 2.14 kg (95% CI = −10.92 to 15.20 kg, P = 0.748, Figure 3A).

### Muscle strength: leg extension

Six studies evaluated the effect of nutritional intervention on muscle strength by comparing leg extension muscle strength between the intervention and placebo groups [17], [19], [20], [26]–[28]. Five of the 6 studies reported that the strength of the leg extension significantly increased in both groups during the duration of the study [8], [19], [26]–[28]. Two studies reported no significant change in the strength of the leg extension versus treatment time or group [17], [20].

Among the 6 studies with protein supplementation, 2 did not provide the mean muscle strength of leg extension for both groups at baseline and at completion of study [8], [17], hence the meta-analysis included 4 studies [20], [26], [28]. Since moderate heterogeneity was found among the studies (Q = 4.52, df = 3, *P* = 0.210; I^2^ = 33.66%), a fixed-effects model was used for the meta-analysis. The difference in mean change from baseline to end of study in the 4 studies ranged from 0 to 18 kg with the overall difference in mean change from baseline to end of study being 2.28 kg (95% CI = −1.73 to 6.29 kg, P = 0.265, Figure 3B). The combined difference in mean change of muscle strength by leg extension from baseline to end of study revealed no significant difference between the control and treatment groups.

### Sensitivity analysis

**Figure 4:**
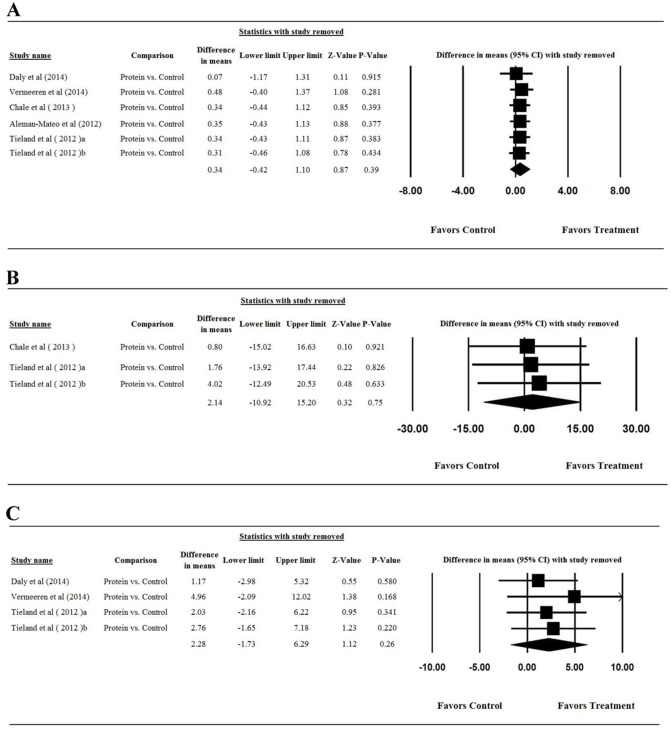
Results of sensitivity analysis to examine the influence of individual studies on pooled estimates as determined using the leave-one-out approach: (A) lean-body-mass; (B) muscle strength of double leg press. Abbreviation: CI, confidence interval.

To assess the effect of a single study on the results of the meta-analysis, we removed each study in turn for LBM (Figure 4A), muscle strength by double leg press (Figure 4B), and muscle strength by leg extension (Figure 4C). The removal of any study did not alter the magnitude and direction; taken together, these results indicated that the meta-analysis showed good reliability.

### Publication Bias

**Figure 5:**
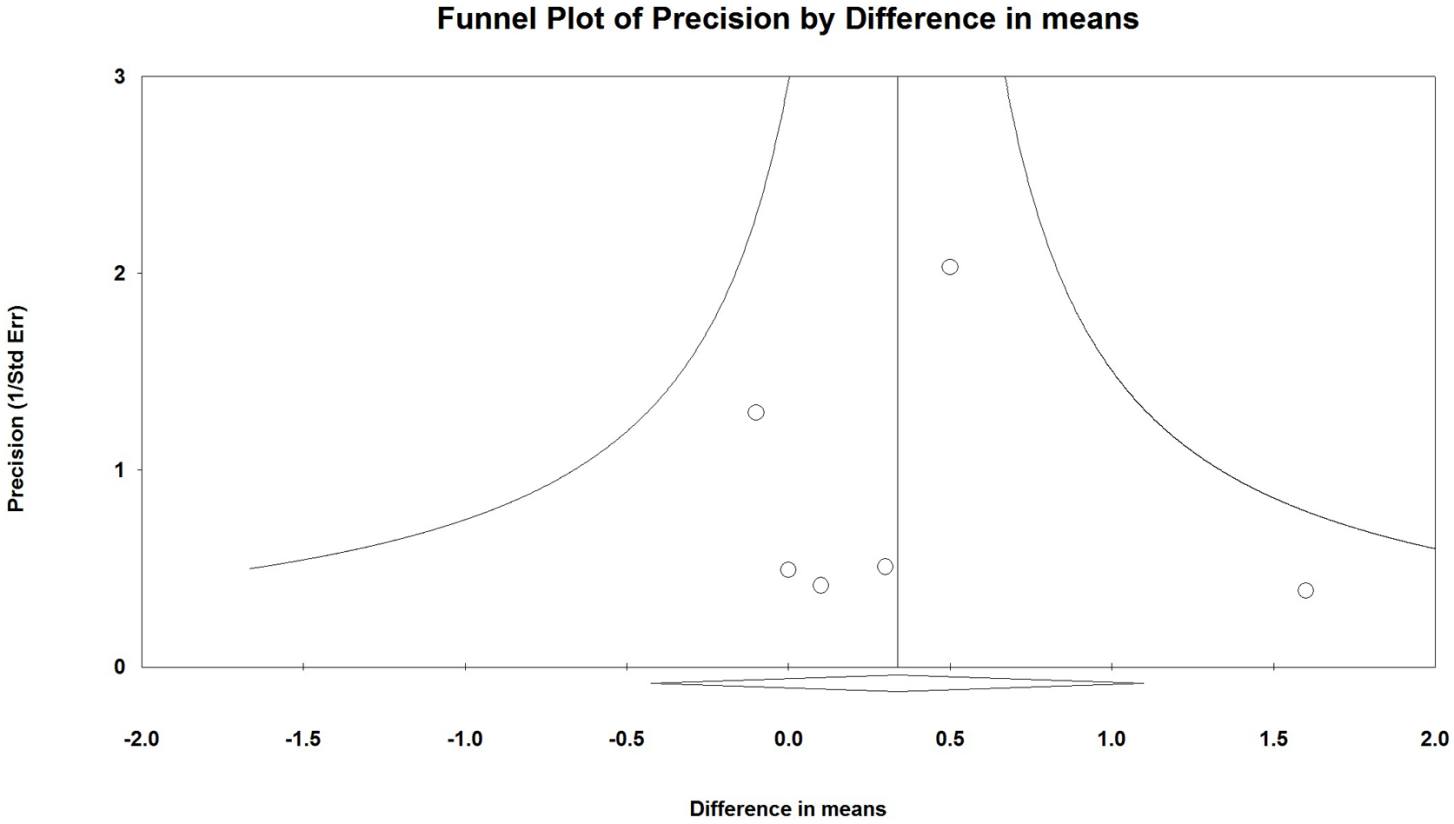
Funnel plot for the assessment of publication bias for studies included in the meta-analysis of the assessment of the mean change from baseline in lean body mass after intervention.

Publication bias (Figure 5) was assessed using the LBM results only as more than 5 studies reported results for this outcome (note: more than five studies are required to detect funnel plot asymmetry [30]). Egger’s test results showed that there was no publication bias in LBM results among studies (Figure 5, t = 0.046, one-tailed P = 0.483).

## Discussion

This meta-analysis of 9 placebo-controlled studies assessed protein and amino acid supplementation on improving LBM in elderly subjects. Our analysis detected no significant differences between placebo and treatment groups in mean change from baseline to the end of the studies of LBM or muscle strength as measured by double leg press or leg extension in a mixed elderly population.

Multiple studies, several of which were included in our meta-analysis, found no significant benefit of protein supplementation compared to placebo in improving LBM [8], [20], [26]–[29], [31]. However, protein supplementation has increased LBM and strength in some studies [32]. This inconsistency raises questions of whether it may be due to differences in study design, difference in efficacy of the supplements tested, or differences among the populations analyzed. Identification of the variables that influence the outcome of high protein intake towards a significant increase in LBM or leg strength would provide important guidance for physicians and for cost effective usage of protein supplementation.

The health and physical status of the patient may influence outcomes. Physical condition may affect response to protein or amino acid supplementation. One study showed that whey supplementation augmented LBM significantly more than placebo in pre-frail and frail elderly subjects receiving resistance training [20] but not in another study of elderly subjects with limited mobility that also received protein supplements and resistance training [8]. These findings suggest that the physical condition of the elderly is not solely responsible for the divergent results. Undernourishment may be another condition that significantly affects the outcome [33]. An earlier meta-analysis showed that protein supplementation induced significant weight gain in undernourished elderly subjects and may reduce mortality [33]. In addition, some elderly subjects may have reduced sensitivity to the amino acid induced anabolic signals and thus have a higher propensity to muscle wasting [21]. Addition of leucine appeared to normalize these anabolic signals [14], [32]. The health status or stage of the skeletal muscle (whether the person does or does not have sarcopenia) may also affect their ability to respond to protein or amino acid supplementation.

The provided supplement or its dosage also may impact treatment outcomes since supplementation with essential amino acid was not as efficacious in increasing LBM in elderly subjects as whey protein in a direct comparison [32]. Both whey and caseinate supplementation induced a similar increase in protein synthesis after heavy resistance training in healthy elderly participants [12]. Interestingly, a fortified, hydrolyzed collagen protein supplement added to a relatively low-protein diet maintained LBM to a greater extent than whey protein [34]. In some studies [7], [11], [13], supplementation with essential amino acids improved LBM or muscle protein synthesis rate in elderly subjects; however, another study did not find any benefit of supplementing with amino acids [14].

Loss of muscle tissue or development of sarcopenia is accelerated by bed rest and lack of physical activity [23]. The elderly in the Tieland et al study [26] performed resistance-type exercise 2 times per week for 24 weeks and had a significant increase in LBM in the supplement group, whereas 5 of the included studies involved participants on bed rest [18], no exercise program [17], [20], [29], or patients who were hospitalized [28]. All participants in the study reported by Daly et al [27] performed resistance training. Consistent with the findings of Tieland et al [26], Daly et al [27] found that participants in supplement group had a significant increase in LBM compared with participants in the control group. The participants of the Chale et al study [8] also performed resistance training and both treatment and placebo groups had similar increases in LBM and leg muscle strength; although, the whey group showed a significant improvement in physical performance [8]. Similarly, in the study by Leenders et al [19] both treatment and control groups reported a mean of 1.55 h physical exercise daily and both groups had similar but significant increases in mean leg strength (both leg press and extension). The resistance training regimen in the study by Tieland et al [26] included several more types of exercises than that of Chale et al [8], while the training regimen in the study of Daly et al [27] involved progressive resistance training. Hence, the beneficial interaction between resistance training and whey protein supplementation on muscle mass and strength gain may depend to some extent on the type of resistance training regimen used. In support for the benefits of concurrent resistance training, a meta-analysis of six studies of older participants reported that protein supplementation augmented loss of fat free mass [35].

There are several limitations to this analysis that should be considered when interpreting the findings. There are a number of outcomes that this analysis did not assess primarily due to limitations of the included studies. These outcomes included (but are not limited to) gender, physical performance and activity, and muscle stage. We also included only RCT. Some non-RCT trials have been done that indicate protein or amino acid supplementation may improve LMB [36]. The relatively small number of included studies, the small subject populations, diverse supplements administered, different outcomes measured and study designs used in the 9 included studies further confounds the analysis. In particular, several studies incorporated exercise (for both intervention and control participants) as part of the study [8], [26], [27], while the others did not. Although our meta-analysis suggests that exercise had little effect on the change in LBM in the individual studies, this possibility clearly warrants examination in appropriately designed studies. In addition, it is not clear whether our findings will be applicable to elderly subjects who receive other types of supplements, had different exercise regimens, or health status than those used in the 9 included studies. The small number of RCTs that address the question of the use of protein or amino acid supplements to reduce muscle loss in elderly subjects highlights the need for more controlled studies to address this medically important question.

In conclusion, these results indicate that amino acid or protein supplements did not increase lean body mass gain and muscle strength significantly more than placebo in a diverse elderly population. The ability of protein or amino acid supplementation to augment muscle mass and strength may depend on the nutritional physical status of the participants, or their ability to digest protein and absorb the amino acids, the sensitivity of the anabolic pathways in muscles, and the resistance training regimen itself.

## Supporting Information

Figure S1 **PRISMA 2009 Flow Diagram.**
(DOC)

Checklist S1 **PRISMA 2009 Checklist.**
(DOC)
