# Supplementary material for: Clinical Effectiveness of Protein and Amino Acid Supplementation on Building Muscle Mass in Elderly People: A Meta-Analysis
Source: PLoS One. 2014 Sep 30;9(9):e109141. doi: 10.1371/journal.pone.0109141 (PMC4182521; doi:10.1371/journal.pone.0109141)
Supplement: Figure S1 — PRISMA 2009 Flow Diagram. (DOC) [file pone.0109141.s001.doc]

**Figure S1**
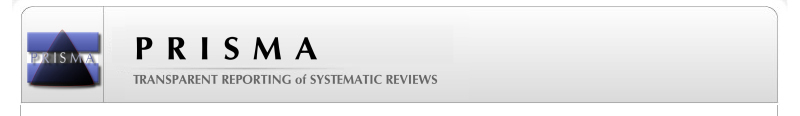
**. PRISMA 2009 Flow Diagram**

**Screening**

**Included**

**Eligibility**

**Identification**

Records identified through database searching
( n = 1175 )

Additional records identified through other sources
( n = 593 )

Records after duplicates removed
( n = 1768 )

Records screened
( n = 1768 )

Records excluded
(n = 1744 )

Full-text articles assessed for eligibility
( n = 24 )

Full-text articles excluded, with reasons
(n = 18 )

Single arm study (1)

Comparison between different amino acid/supplement interventions (8)

Outcome not interest (9)

Studies included in qualitative synthesis
( n = 6 )

Studies included in quantitative synthesis (meta-analysis)
( n = 6 )
